# Plasmid pEC156, a Naturally Occurring *Escherichia coli* Genetic Element That Carries Genes of the EcoVIII Restriction-Modification System, Is Mobilizable among Enterobacteria

**DOI:** 10.1371/journal.pone.0148355

**Published:** 2016-02-05

**Authors:** Olesia Werbowy, Tadeusz Kaczorowski

**Affiliations:** Department of Microbiology, University of Gdansk, Wita Stwosza 59, Gdansk, Poland; University of Manchester, UNITED KINGDOM

## Abstract

Type II restriction-modification systems are ubiquitous in prokaryotes. Some of them are present in naturally occurring plasmids, which may facilitate the spread of these systems in bacterial populations by horizontal gene transfer. However, little is known about the routes of their dissemination. As a model to study this, we have chosen an *Escherichia coli* natural plasmid pEC156 that carries the EcoVIII restriction modification system. The presence of this system as well as the *cis*-acting *cer* site involved in resolution of plasmid multimers determines the stable maintenance of pEC156 not only in *Escherichia coli* but also in other enterobacteria. We have shown that due to the presence of *oriT*-type F and *oriT*-type R64 loci it is possible to mobilize pEC156 by conjugative plasmids (F and R64, respectively). The highest mobilization frequency was observed when pEC156-derivatives were transferred between *Escherichia coli* strains, *Enterobacter cloacae* and *Citrobacter freundii* representing coliform bacteria. We found that a pEC156-derivative with a functional EcoVIII restriction-modification system was mobilized in enterobacteria at a frequency lower than a plasmid lacking this system. In addition, we found that bacteria that possess the EcoVIII restriction-modification system can efficiently release plasmid content to the environment. We have shown that *E*. *coli* cells can be naturally transformed with pEC156-derivatives, however, with low efficiency. The transformation protocol employed neither involved chemical agents (e.g. CaCl_2_) nor temperature shift which could induce plasmid DNA uptake.

## Introduction

Plasmids are extrachromosomal mobile genetic elements that are part of the genetic content of almost all prokaryotes examined so far. Although non-essential to microorganisms, they can provide the host with a useful cargo of genes important for adaptation to diverse and changing environmental conditions [1–4]. Among these beneficial genes, a special role is played by those that constitute restriction-modification (RM) systems that combine the activity of two enzymes: a restriction endonuclease and a cognate DNA methyltransferase. Their primary role relies on protecting bacteria against phage invasion [5]. However, other functions such as the involvement of RM systems in genetic recombination, genetic variation, speciation and others that can increase the host fitness are also considered [6–9]. When present in cells, the RM systems, apart from their aforementioned diverse functions, may also modulate the flow of incoming DNA molecules [10–12]. As such, they can be considered as key elements that can control circulation of genetic determinants in the environment. Due to their structural and functional diversity, the RM systems can be grouped into four distinct types. While the majority of RM systems are located on bacterial chromosomes, some of them, especially those representing type II can be found in naturally occurring plasmids. This may facilitate dissemination of these genetic elements among bacteria by means of horizontal gene transfer as suggested by bioinformatic analyses [13]. However, in depth examination of 2261 prokaryote genomes revealed that RM systems are rare in plasmids and the host spectrum for such plasmids is rather narrow [14]. This raises the following questions: (i) are there any specific constraints that prevent spread of plasmid borne RM systems among bacteria; and (ii) how efficient is horizontal transfer of such plasmids?

**Fig 1:**
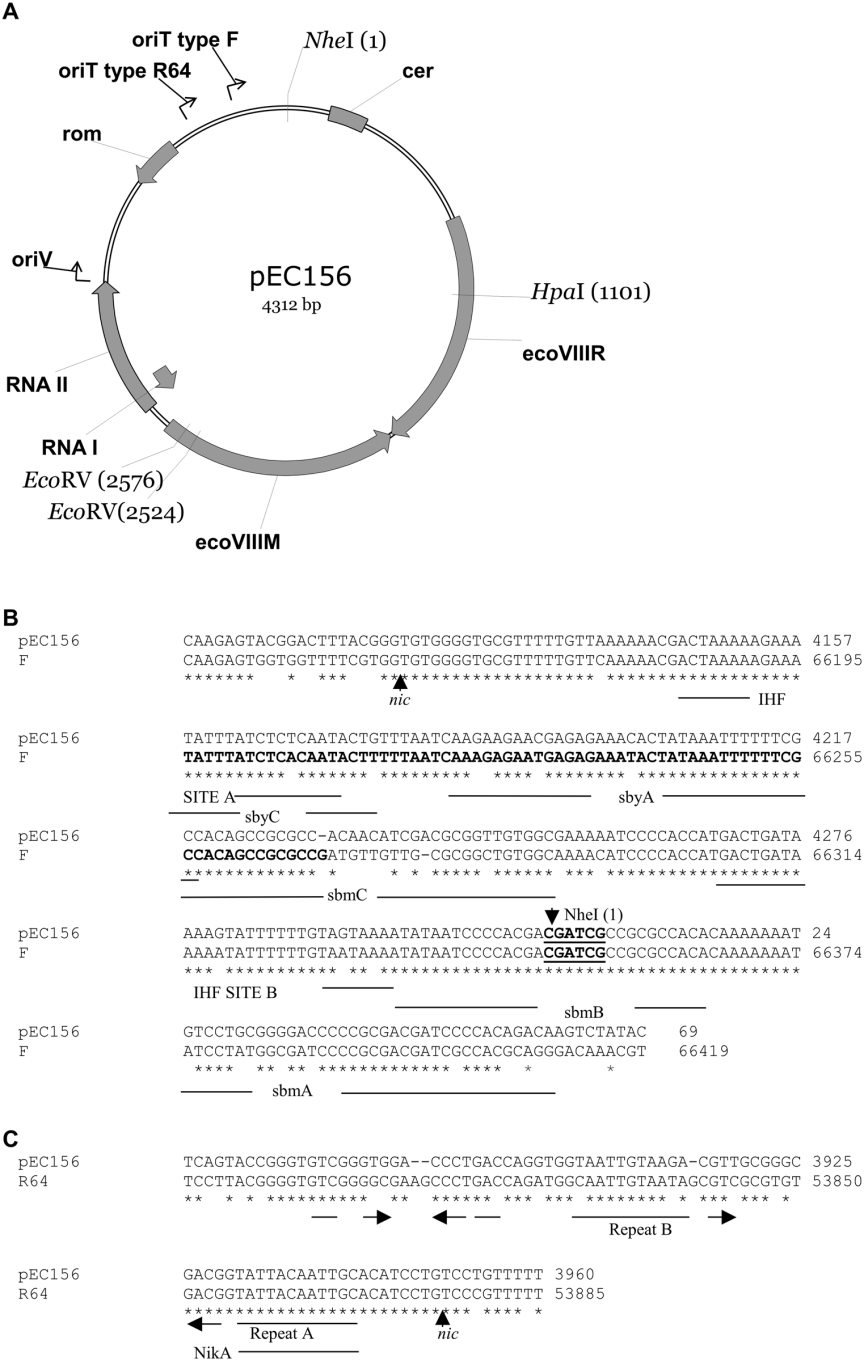
A map of plasmid pEC156 (**A**). The genes coding for the EcoVIII RM system, *cer* locus, *rom* gene as well as regions with *oriT* F-like, *oriT* R64-like sequences and genes that are engaged in the priming (RNA II) and controlling the initiation of plasmid DNA replication (RNA I) are indicated. Alignment of the pEC156 nucleotide sequence with *oriT* of F plasmid (**B**) and *oriT* of R64 plasmid (**C**). The minimal region that allows *oriT* F-dependent plasmid mobilization is in boldface. The position of nick sites (*nic*) are indicated. Binding sites for plasmid F TraM (sbmA, sbmB and sbmC), TraY (sbyA and sbyC) and IHF protein as well as NikA binding site on plasmid R64 are underlined. Imperfect inverted repeats are indicated by arrows. Asterisks indicate identical nucleotides. The accession numbers of nucleotide sequences of plasmids pEC156, F and R64 that have been deposited in the Genbank database are AF158026, AP001918 and AB027308, respectively.

As a model in our studies we have chosen the naturally occurring plasmid pEC156 of *Escherichia coli* E158568 (serotype O156; [15]) that is a ColE1-type replicon [16]. It includes an origin of replication and two untranslated genes coding for RNA I and RNA II molecules, both involved in plasmid DNA replication (Fig 1A). Further, analysis of the pEC156 nucleotide sequence revealed a lack of the *mob* genes, but the presence of two loci with similarity to *oriT* of plasmid F (*E*. *coli*) and *oriT* of plasmid R64 (*Salmonella typhimurium)*, respectively (Fig 1B and 1C). Each of them represents different incompatibility group, IncFI and IncI1, respectively. Plasmids that carry the *oriT* locus can be efficiently mobilized by self-transmissible conjugative plasmids such as F or R64 [17–20]. pEC156 contains genes coding for EcoVIII, a type II RM system comprising a site-specific restriction endonuclease and DNA methyltransferase that recognize the specific palindromic sequence 5’-AAGCTT-3’ [21]. Computational analysis of the pEC156 nucleotide sequence revealed the presence of a specific locus showing a pronounced nucleotide sequence similarity to the *cer* locus (ColE1 resolution) of plasmid ColE1. When present, the *cis*-acting *cer* site ensures stable inheritance of the ColE1-type replicons by random partition increasing the probability that at cell division each daughter cell receives at least one copy of the plasmid [22]. This locus contains binding sites for the XerC and XerD recombinases [23, 24] and regions that interact with the ArgR and PepA proteins [25–27]. All four proteins are host-encoded and mediate conversion of plasmid multimers that arise by homologous recombination to monomers. Our previous work demonstrated that three factors ensure stable maintenance of pEC156 in *E*. *coli* and other enterobacteria: (i) a *cis*-acting *cer* site involved in resolution of plasmid multimers, (ii) a gene coding for EcoVIII endonuclease, and (iii) plasmid copy number control [28]. In the same report we also showed that pEC156 can be stably maintained in members of the *Enterobacteriaceae* family. This is based on a mechanism by which descendants of cells that have lost the plasmid encoding a RM system cannot survive due to a reduced pool of DNA methyltransferase molecules. Lack of sufficient protection of the genomic DNA against the action of cognate restriction endonuclease leads directly to bacterial cell death [29, 30]. Such mechanisms, based on postsegregational cell killing, are also typical for other toxin-antitoxin modules that participate in maintenance of many bacterial plasmids [31, 32].

In the present study we focused on routes of dissemination that enable transfer of the pEC156 plasmid among enterobacteria. We designed and performed experiments providing direct evidence that such transfer occurs through mostly conjugal machinery and to a lesser extent by natural transformation. In addition, we also investigated the effect of the EcoVIII RM system on plasmid DNA release from bacterial cells. As a result, we found that bacteria that possess an active EcoVIII RM system can efficiently release their plasmid content to the environment.

## Materials and Methods

### Strains and plasmids

The following strains of bacteria were used in this work: *Escherichia coli* HV1735 (F’_ts_114*lac*::Tn*5*; Km^R^) as a source of conjugal F-plasmid was obtained from Dr. George Szatmari (University of Montreal); R64*drd*11 conjugal plasmid that confers tetracycline (Tc) and streptomycin (Sm) resistance to the cell was obtained from Dr. Pierre Cornelis (Vrije Universiteit Brussel); *E*. *coli* DH5α Rif rifampicin resistant (Rif^R^) derivative of DH5α [33] was obtained from Dr. Piotr Zaleski (Institute of Biotechnology and Antibiotics, Warsaw); *E*. *coli* MG1655Δ*lac* (Fˉ Δ*lac*::Tn*10* (*zah*281) (Tc^R^) was obtained from Dr. Marian Sektas (University of Gdansk). *E*. *coli* MG1655 (wild type), *E*. *coli* HB101 (F^−^ *recA*13 *rpsL*20(Sm^R^), *Klebsiella oxytoca* KPD118-BA, *Citrobacter freundii* NCTC 9750, *Salmonella enteritidis*, *Enterobacter cloacae* ATCC 13047 (Ap^R^, Sm^R^) were obtained from the Collection of Plasmids and Microorganisms, University of Gdansk, Gdansk, Poland. All bacteria were cultivated in Luria broth (LB) or Luria agar (LA) plates [34] at 37°C. When necessary appropriate antibiotics were used in the following concentrations: chloramphenicol (Cm) 30 μg ml^-1^, kanamycin (Km) 50 μg ml^-1^, ampicillin (Ap) 100 μg ml^-1^, tetracycline (Tc) 15 μg ml^-1^, rifampicin (Rif) 50 μg ml^-1^ and streptomycin (Sm) 25 μg ml^-1^. Plasmids pIB8 (EcoVIII R^+^M^+^ *cer*^+^ *rom*^+^ Cm^R^, 5.2-kb) and pIB9 (EcoVIII R^−^M^−^ *cer*^+^ *rom*^+^ Cm^R^, 3.7-kb) are derivatives of pEC156 [16] and were obtained from Dr. Iwona Mruk (University of Gdansk, Poland). Plasmid pIB8 was constructed by cloning a 0.8-kb XbaI fragment carrying an antibiotic resistance cassette for chloramphenicol taken from pKRP10 [35], into pEC156 linearized with NheI [16]. Plasmid pIB9 was constructed by deleting from pIB8 a 1.48-kb HpaI-EcoRV DNA fragment carrying EcoVIII RM system [16]. Plasmid pOB9Cm is a pEC156-derivative lacking the two loci with similarities to *oriT* of plasmid F and *oriT* of plasmid R64 and was constructed by replacing a 1.4-kb BamHI cassette conferring resistance to kanamycin in pOB9 (EcoVIII R^+^M^+^ *cer*^+^ *rom*^−^ Km^R^; [28]) with a 0.9-kb BamHI cassette carrying chloramphenicol resistance gene derived from pKRP10 [35]. Plasmid pIB8A and pIB9A were constructed by cloning into the SalI site of pIB8 or pIB9 a cassette (1.5-kb) carrying the ampicillin resistance gene derived from pUC19 [36]. This cassette was obtained by PCR-based DNA amplification using primers Am1 (5’CTCGTCGACATCTGCTCTGATGCCGCATAG3’) and Am2 (5’CGGGTCGACAGTTGGTAGCTCTTGATCCGGCA3’). Standard protocols were used for molecular cloning [34, 37]. Plasmid pACYC177 (Ap^R^, Km^R^; [38]) was used in the plasmid release experiment and pBR322 (Ap^R^, Tc^R^; [39]) was used in the plasmid mobilization experiment for selection of transconjugants. The EcoVIII R^+^ phenotype was assayed according to the method described previously [40]. Plasmids constructed in this study (pIB8A, pIB9A) were deposited in the Collection of Plasmids and Microorganisms, University of Gdansk, Gdansk, Poland.

### Plasmid mobilization assay

The donor bacteria carried either the F’_ts_114*lac*::Tn*5* (Km^R^) or R64*drd*11 (Tc^R^) conjugative plasmid. The recipient bacteria are listed in S1 Table where the details concerning mating pairs are outlined. The conjugation protocol used in this study was essentially as described by Rehel and Szatmari [41]. In plasmid mobilization experiments two sets of donor bacteria were used, one using the pEC156-derivative pIB8 (EcoVIII R^+^M^+^ Cm^R^) and another with pIB9 (EcoVIII R^−^M^−^ Cm^R^). In these experiments the recipient bacteria carried for counterselection pBR322 that confers resistance to ampicillin and tetracycline. Plasmid mobilization was performed as follows: overnight cultures of donor and recipient bacteria were diluted in LB-broth (1/40 and 1/20 dilution, respectively) and were grown to log-phase and mixed (0.8:0.5, donor and recipient, respectively, v/v). The mixtures were cultivated for 2 h with shaking (30 rpm). Finally, transconjugants were selected on LB agar plates supplemented with Cm+Ap+Tc to estimate the mobilization frequency of pEC156-derivatives and plates supplemented with Km+Ap+Tc were used to estimate the frequency of F’_ts_114*lac*::Tn*5* conjugal transfer (S1 Table). The presence and size of transferred plasmids were verified by restriction analysis. No rearrangements were observed. Mobilization efficiency was calculated as ratio of mobilization frequency to the frequency of conjugative plasmid transfer. For experiments where only plasmid mobilization was observed but not conjugative plasmid transfer, frequencies of mobilization were calculated. All experiments were performed in triplicate and repeated three times.

### Plasmid release

To measure plasmid release *E*. *coli* MG1655 carrying plasmid pACYC177 alone or in combination with pIB8 (EcoVIII R^+^M^+^) or pIB9 (EcoVIII R^−^M^−^) was diluted in LB medium (1/10 000 dilution) to a final concentration 1–4 ×10^5^ cells ml^-1^ and grown at 37°C for 12 h. Aliquots were taken every hour to determine the titer of the bacteria and optical density of the culture. At the same time 20 ml samples of bacterial culture were screened for the presence of released plasmid DNA. First, the bacteria were harvested by centrifugation (10 min, 2000 × *g*) under mild conditions to prevent cell lysis. The resulting supernatant was then passed through a syringe nitrocellulose filter (0.22 μm) followed by standard precipitation of plasmid DNA with isopropanol [34]. The obtained plasmid DNA was then introduced into *E*. *coli* HB101 (Sm^R^) competent cells using CaCl_2_ standard procedure [34] and plated on LA agar plates supplemented with Ap and Sm to select for transformed bacteria carrying pACYC177. The efficiency of plasmid release was determined for each sample taken as a ratio of transformants (c.f.u.) to calculated titer of the host. The results obtained were normalized against bacteria that carried only pACYC177. All experiments were performed in triplicate and repeated three times.

### *E*. *coli* natural transformation

In this work we used a protocol developed by others [42]. Briefly, an overnight culture of *E*. *coli* HB101 was diluted in LB medium (1/100 dilution) and bacteria were grown to stationary-phase. Then, aliquots (50 μl) of culture were transferred to Eppendorf tubes and left open under sterile conditions for 12 h at 37°C without shaking. In the next step, 2 μg of plasmid DNA [pIB8A (EcoVIII R^+^M^+^, Ap^R^), pIB9A (EcoVIII R^−^M^−^, Ap^R^) or pUC19 (Ap^R^) as control plasmid] was added to each tube. Then bacteria were spread on LB agar plates supplemented with antibiotic (Ap). Plasmids were isolated from transformants and verified by restriction analysis. This transformation protocol neither involved chemical agents (e.g. CaCl_2_) nor temperature shift which can induce plasmid DNA uptake. Transformation efficiency was calculated as the number of antibiotic resistant colonies per pmol of plasmid used. All experiments were performed in triplicate and repeated three times.

### Statistical analysis

To assess statistical significance two tailed Student unpaired *t* test was used with GraphPad Prism 5.0 software (GraphPad Software) with a *P* value <0.05 (95% confidence interval).

## Results

### pEC156 possesses two functional origins of transfer

Bioinformatic analysis of nucleotide sequence of pEC156 revealed presence of two loci with similarities to the origin of transfer (*oriT*) of the *E*. *coli* self-transmissible conjugative plasmids, F (coordinates 4098-4312/1-69; Fig 1B) and R64 (coordinates 3867–3960, Fig 1C). The first sequence which we designated as *oriT*-type F of plasmid pEC156, encompasses 274 bp and shows high identity (86.5%) to the *oriT* locus of the F-plasmid (coordinates 66136–66410, GenBank AP001918). This site, located upstream of the *traM* gene is essential for F-plasmid conjugal transfer due to the presence of a relaxation site (*nic* site) that when nicked initiates the transfer of single strand of DNA into the recipient in the 5’→3’ direction. This complex locus also contains binding sites for the TraM (sbmA, sbmB and sbmC; Fig 1B), TraY (sbyA and sbyC) and IHF proteins. It was shown that while the TraM protein is essential for F-plasmid conjugal transfer, it is not required for ColE1 mobilization [43]. The second region of pEC156 which was termed as *oriT*-type R64, is 93-bp in length and is similar (83.9% identity) to the *oriT* locus of plasmid R64 (coordinates 53792–53885, GenBank AB027308). Bioinformatic analysis revealed that the R64 *oriT* highly conserved core nucleotide sequence, which is required for plasmid transfer (nick region and repeat A; [44]), is almost identical to its corresponding region of pEC156 (Fig 1C). Moreover, both aforementioned pEC156 *oriT* loci share a pronounced similarity to corresponding sequences of other enterobacterial plasmids such as pColG (*E*. *coli*; GenBank DQ286390), pKPN2 (*K*. *pneumoniae*; GenBank AF300473) carrying a type II RM system, pSe-Kan (*S*. *enterica* subsp. *enterica* serovar Typhimurium; GenBank HQ230976), and 2-mD plasmid (*Shigella flexneri*; GenBank M25995).

The calculated efficiency of pIB8 (EcoVIII R^+^M^+^) mobilization by F’_ts_114*lac*::Tn*5* and R64*drd*11 was 0.55±0.23 and 0.24±0.12, respectively (Table 1). The lower mobilization efficiency in case of R64-plasmid might be explained by the fact that this plasmid is not native to *E*. *coli*. The host for R64 plasmid is *Salmonella typhimurium*. The values obtained are close to those reported for the mobilization of the pKPN2 plasmid that carries genes of the Kpn2I RM system [45]. Although pKPN2 contains the *oriT* loci with similarity to conjugative plasmids F and R64, it can be mobilized by conjugative plasmid F’(*lac-gal*) but not by R64*drd*11 [45]. In addition, we found the transfer of the pEC156-derivative with a non-functional EcoVIII RM system (pIB9; (EcoVIII R^−^M^−^) to be as effective as pIB8 transmission, with a mobilization efficiency of F’_ts_114*lac*::Tn*5* and R64*drd*11 to be 0.63±0.29 and 0.52±0.19, respectively (Table 1). The results obtained using pIB8 and pIB9 suggest that both origins of transfer are functional in plasmid pEC156 mobilization. In a control experiment, we used a pEC156-derivative deprived of both *oriT* loci (pOB9Cm). We observed that this derivative was not mobilized by the conjugative plasmids used in this study (Table 1). Since the observed efficiency of mobilization of pIB8 and pIB9 was higher for plasmid F’_ts_114*lac*::Tn*5* than R64, and since we wanted to use plasmids native to *E*. *coli* in the following experiments on pEC156 mobilization the aforementioned F-plasmid was used.

**Table 1:** Mobilization of pEC156 derivatives by IncFI and IncI1 conjugative plasmids.

Test plasmid	Conjugative plasmid	Mobilization efficiency
pIB8 (R^+^M^+^ *oriT* F^+^ *oriT* R64^+^)	F’ts114*lac*::Tn*5*	0.55±0.23
pIB8 (R^+^M^+^ *oriT* F^+^ *oriT* R64^+^)	R64*drd*11	0.24±0.12
pIB9 (R^-^M^-^ *oriT* F^+^ *oriT* R64^+^)	F’ts114*lac*::Tn*5*	0.63±0.29
pIB9 (R^-^M^-^ *oriT* F^+^ *oriT* R64^+^)	R64*drd*11	0.52±0.19
pOB9Cm (R^+^M^+^ *oriT* F^-^ *oriT* R64^-^)	F’ts114*lac*::Tn*5*	0.0001±0.0001
pOB9Cm (R^+^M^+^ *oriT* F^-^ *oriT* R64^-^)	R64*drd*11	0.0007±0.0002

The mobilization efficiency is expressed relative to the frequency of transfer of the conjugative plasmid. *E*. *coli* HB101 and *E*. *coli* DH5α Rif were used, as donor and recipient, respectively.

### Analysis of pEC156-derivatives mobilization by plasmid F

**Fig 2:**
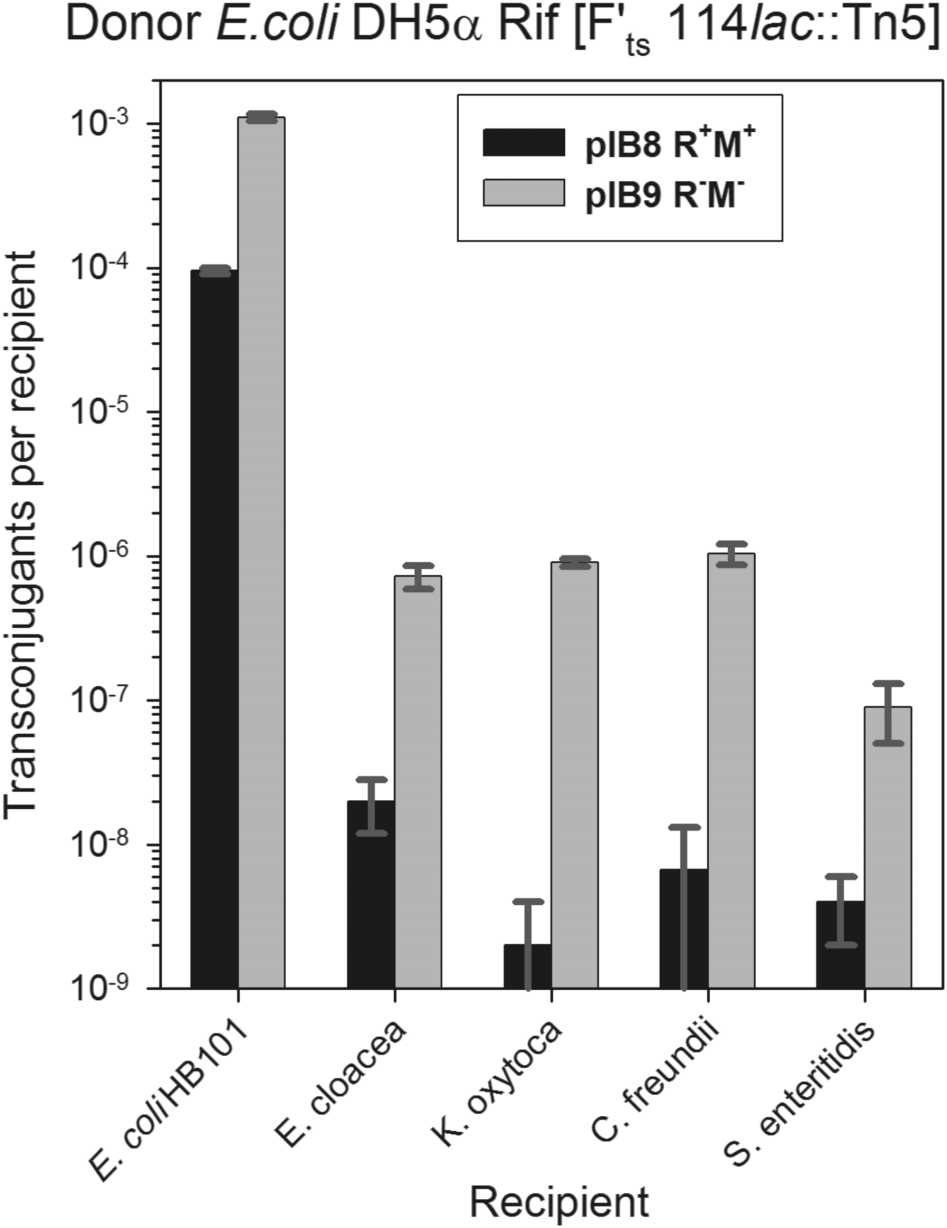
The effect of the presence of EcoVIII RM system in recipient cells on efficiency of conjugal transfer of plasmid F. *E*. *coli* DH5α Rif [F’_ts_114*lac*::Tn*5*, Km^R^] was used as donor. The following recipient bacteria were assayed: *Escherichia coli* HB101, *Enterobacter cloacae*, *Klebsiella oxytoca*, *Citrobacter freundii*, and *Salmonella enteritidis* that carried either pEC156-derivative pIB8 (EcoVIII R^+^M^+^, black bars) or pIB9 (EcoVIII R^−^M^−^, grey bars). Each column represents the mean value (± standard deviation) from three repeats. Statistical analysis revealed correlation between presence of a plasmid with RM system in the recipient bacteria and frequency of F plasmid conjugal transfer (*P*<0.0007 for *E*. *coli*, *E*. *cloacae*, *K*. *oxytoca*, *C*. *freundii*; and *P* = 0.02 for *S*. *enteritidis*; Student *t* test).

Conjugation experiments were performed between *E*. *coli* DH5α Rif [F’_ts_114*lac*::Tn*5*, Km^R^] and the following recipient bacteria carrying plasmid pIB8 (EcoVIII R^+^M^+^ Cm^R^) or pIB9 (EcoVIII R^−^M^−^ Cm^R^): *E*. *coli* HB101, *E*. *cloacae*, *K*. *oxytoca*, *C*. *freundii* and *S*. *enteritidis*. The bacteria used represent closely related species [46]. We found that the activity of the EcoVIII restriction endonuclease in recipient cells (bacteria with pIB8) affected the efficiency of transconjugant formation. This is not surprising since the F-plasmid possesses 14 EcoVIII restriction sites. In the case of *E*. *coli* HB101 [pIB8 EcoVIII R^+^M^+^] the frequency was 9.5± 0.5 × 10^−5^ transconjugants per recipient cell (Fig 2), while for the same strain carrying pIB9 (EcoVIII R^−^M^−^) this value was about 10-fold higher (1.1± 0.1 × 10^−3^; Fig 2). A similar effect was observed with the conjugal transfer of the F’_ts_114*lac*::Tn*5* plasmid to other enterobacteria that carried pIB8 or pIB9 (Fig 2). Statistical analysis revealed correlation between presence of a plasmid with RM system in the recipient bacteria and frequency of F-plasmid conjugal transfer (*P*<0.0007 for *E*. *coli*, *E*. *cloacae*, *K*. *oxytoca*, *C*. *freundii*; and *P* = 0.02 for *S*. *enteritidis*; Student *t* test). The results obtained are consistent with recently reported findings that show that the presence of a RM system affects, but is not an absolute barrier for conjugation [12].

The transconjugants obtained from this initial mating, that carried a conjugal plasmid and a pEC156 derivative (pIB8 or pIB9), were used in further studies. The aim of these experiments was to investigate the pEC156-derivative mobilization by the conjugative F’_ts_114*lac*::Tn*5* plasmid as it is outlined in S1 Table. Plasmid mobilization was performed as described in Materials and methods. Results obtained on the frequency of pEC156-derivatives mobilization and frequency of the F’_ts_114*lac*::Tn*5* plasmid conjugal transfer are presented in Tables 2 and 3. They indicate that pEC156 derivatives pIB8 and pIB9 can be mobilized by the conjugative plasmid and transferred from the host to *E*. *coli* and other enterobacteria at different frequencies. In general, the frequency of pIB9 mobilization was significantly higher due to the lack of a functional EcoVIII RM system (Table 3). The rate of DNA transfer during conjugation is approximately 750 nucleotides per second at 37°C [47]. In this respect we should not observe differences in mobilization frequencies due to the plasmids size as they are relatively small (5.2 and 3.7-kb for pIB8 and pIB9, respectively). Therefore, we assume that the most important factor that affects plasmid transmission is the presence of functional restriction-modification system. In case of the *E*. *coli* strains tested, we observed a high frequency of mobilization (10^−3^–10^−5^) either for the wild type strain MG1655Δ*lac* and HB101. The last strain is defective in recombination (*recA* mutant). In these experiments, when plasmids were transferred between *E*. *coli* strains, there was almost no difference in mobilization frequency whether the pIB8 (EcoVIII R^+^M^+^) or the pIB9 (EcoVIII R^−^M^−^) plasmid was used. The pEC156 derivatives were also mobilized from *E*. *coli*, however at a noted lower frequency, to other enterobacteria. The highest frequency was observed for transfer to *K*. *oxytoca* (10^−6^–10^−7^), *E*. *cloacae* (10^−7^) and *C*. *freundii* (10^−7^) representing the coliform bacteria. The lowest mobilization frequency was experienced for pIB8/pIB9 transfer from *E*. *coli* to *S*. *enteritidis* (10^−9^). As plasmid mobilization from enterobacteria other than *E*. *coli* is concerned the highest frequency was observed for transfer between *C*. *freundii* and *E*. *coli* HB101 (pIB9, 10^−5^). Similar frequency was observed for transfer between *S*. *enteritidis* and *E*. *coli* MG1655Δ*lac* (pIB9, 10^−5^) and *E*. *cloacae* and *E*. *coli* strains HB101 and MG1655Δ*lac* (pIB9, 10^−5^). In case of the mating pairs *C*. *freundii*-*E*. *coli* HB101 and *S*. *enteritidis*-*E*. *coli* MG1655Δ*lac*, no mobilization was observed for pIB8, while this plasmid was transferred efficiently from *E*. *cloacae* to *E*. *coli* HB101 (10^−5^). In general, the observed mobilization frequencies were lower than frequencies of conjugal transfer of the F’_ts_114*lac*::Tn*5* plasmid (Tables 2 and 3). We have tried to increase pEC156-derivative mobilization frequency by using as recipients bacteria carrying a plasmid with a gene coding for EcoVIII methyltransferase, to ensure a specific methylation pattern prior plasmid transfer. As a result, we observed an increase in mobilization frequency by a factor of 2 (data not shown).

**Table 2:** Mobilization frequency (italics) of pEC156-derivative pIB8 (EcoVIII R^+^M^+^).

		Donor R^+^M^+^				
		*E*. *coli* DH5α Rif [F’, pIB8]	E. cloacae *[F’*, *pIB8]*	*K*. *oxytoca* [F’, pIB8]	*C*. *freundii* [F’, pIB8]	*S*. *enteritidis* [F’, pIB8]
Recipient	*E*. *coli* HB101 [pBR322]	*8*.*4 ± 4 ×10*^*−4*^	*1*.*8 ± 0*.*2 ×10*^*−5*^ *	*<1*.*8 ×10*^*−9*^	*< 1*.*8 ×10*^*−9*^ *	*< 1*.*8 ×10*^*−9*^ *
		**2.2± 1.5 ×10**^**−3**^	**4.6 ± 0.4 ×10**^**−5**^	**< 1.8 ×10**^**−9**^	**1.8 ± 0.3 ×10**^**−7**^ *	**2.6 ± 2.6 ×10**^**−9**^ *
	*E*. *coli* MG1655Δ*lac* [pBR322]	*2*.*5 ± 0*.*2 ×10*^*−4*^	*5*.*6 ± 3*.*2 ×10*^*−6*^ *	*< 1*.*1 ×10*^*−9*^	*< 1*.*1 ×10*^*−9*^ *	*< 1*.*1 ×10*^*−9*^ *
		**2.3 ± 0.2 ×10**^**−4**^	**1.3 ± 0.4 ×10**^**−6**^ *	**< 1.1 ×10**^**−9**^	**3.0 ± 0.3 ×10**^**−8**^ *	**1.8 ± 1.8 ×10**^**−8**^ *
	*E*. *cloacae* [pBR322]	*2*.*5 ± 0*.*7 ×10*^*−7*^	*3*.*2 ± 0*.*4 ×10*^*−7*^ *	*5*.*6 ± 1*.*6 ×10*^*−8*^ *	*9*.*2 ± 3*.*9 ×10*^−9^*	*3*.*0 ± 1*.*5 ×10*^*−8*^ *
		**3.7 ± 0.7 ×10**^**−7**^	**6.2 ± 0.5 ×10**^**−7**^ *	**1.3 ± 0.4 ×10**^**−9**^ *	**1.0 ± 0.03 ×10**^**−7**^	**< 1.0 ×10**^**−9**^ *
	*K*. *oxytoca* [pBR322]	*1*.*8 ± 0*.*9 ×10*^*−6*^ *	*2*.*0 ± 1*.*9 ×10*^*−7*^ *	*8*.*7± 2*.*3 ×10*^*−8*^ *	*2*.*2 ± 0*.*3 ×10*^*−8*^ *	*6*.*7 ± 1*.*3 ×10*^*−8*^ *
		**1.63 ± 0.5 ×10**^**−8**^ *	**3.0 ± 2.6 ×10**^−8^*	**1.3 ± 1.3 ×10**^**−9**^ *	**2.6 ± 0.1 ×10**^**−8**^ *	**< 1.1 ×10**^**−9**^ *
	*C*. *freundii* [pBR322]	*1*.*64 ± 0*.*9 ×10*^*−7*^ *	*1*.*0 ± 0*.*2 ×10*^*−7*^ *	*4*.*6 ± 2 ×10*^*−8*^	*1*.*9 ± 0*.*6 ×10*^*−7*^ *	*8*.*0 ± 3*.*8 ×10*^*−8*^ *
		**< 1.8 ×10**^**−9**^	**< 1.8 ×10**^**−9**^	**< 1.8 ×10**^**−9**^ *	**1.0 ± 0.2 ×10**^**−6**^	**< 1.8 ×10**^**−9**^ *
	*S*. *enteritidis* [pBR322]	*3*.*0 ± 1*.*1 ×10*^*−9*^	*2*.*2 ± 1*.*2 ×10*^*−7*^ *	*1*.*0 ± 1 ×10*^*−8*^	*< 1*.*6 ×10*^*−9*^ *	*< 1*.*6 ×10*^*−9*^ *
		**< 1.6 ×10**^**−9**^	**1.4 ± 1.1 ×10**^**−8**^	**< 1.6 ×10**^**−9**^ *	**< 1.6 ×10**^**−9**^ *	**1.3 ± 1.2 ×10**^**−6**^

The frequency of F-plasmid conjugal transfer is given in boldface. Mobilization frequency/conjugal transfer frequency is the number of transconjugants per recipient. Shown are mean values from 3 independent experiments, and standard deviations are indicated. Asterisks next to the specific values in Table 2 indicate statistically significant differences between mobilization of pIB8 and pIB9 (*P*<0.03; Student *t* test).

**Table 3:** Mobilization frequency (italics) of pEC156-derivative pIB9 (EcoVIII R¯M¯).

		Donor R¯M¯				
		*E*. *coli* DH5α Rif [F’, pIB9]	*E*. *cloacae* [F’, pIB9]	*K*. *oxytoca* [F’, pIB9]	*C*. *freundii* [F’, pIB9]	*S*. *enteritidis* [F’, pIB9]
Recipient	*E*. *coli* HB101 [pBR322]	*8*.*8 ± 6*.*1×10*^*−4*^	*4*.*0 ± 0*.*1 ×10*^*−5*^	*<1*.*8 ×10*^*−9*^	*1*.*3 ± 0*.*09×10*^*−5*^	*1*.*0 ± 0*.*5 ×10*^*−6*^
		**1.6± 0.5 ×10**^**−3**^	**4.6 ± 0.4 ×10**^**−5**^	**< 1.8 ×10**^**−9**^	**2.4 ± 1.8 ×10**^**−5**^	**7.9 ± 4 ×10**^**−8**^
	*E*. *coli* MG1655Δ*lac* [pBR322]	*1*.*4 ± 0*.*7 ×10*^*−4*^	*1*.*3 ± 0*.*3 ×10*^*−5*^	*< 3*.*0 ×10*^*−8*^	*1*.*4 ± 0*.*7 ×10*^*−6*^	*1*.*0 ± 0*.*02 ×10*^*−5*^
		**4.2 ± 2.5 ×10**^**−4**^	**1.2 ± 0.9 ×10**^**−5**^	**1.4 ± 1.4 ×10**^**−9**^	**2.6 ± 1.5 ×10**^**−6**^	**7.6 ± 0.2 ×10**^**−7**^
	*E*. *cloacae* [pBR322]	*3*.*3 ± 0*.*5 ×10*^*−7*^	*5*.*4 ± 2*.*2 ×10*^*−7*^	*2*.*5 ± 0*.*8 ×10*^*−7*^	*7*.*1 ± 3*.*4 ×10*^*−7*^	*2*.*9 ± 1*.*8 ×10*^*−6*^
		**6.3 ± 1.5 ×10**^**−7**^	**1.4 ± 0.5 ×10**^**−6**^	**2.9 ± 0.4 ×10**^**−7**^	**1.0 ± 0.4 ×10**^**−7**^	**1.9 ± 1.5 ×10**^**−7**^
	*K*. *oxytoca* [pBR322]	*2*.*2 ± 0*.*3 ×10*^*−7*^	*3*.*9 ± 2*.*6 ×10*^*−6*^	*4*.*8± 0*.*5 ×10*^*−7*^	*1*.*6 ± 0*.*5 ×10*^*−6*^	*3*.*8 ± 0*.*8 ×10*^*−6*^
		**4.5 ± 1 ×10**^**−6**^	**1.4 ± 0.8 ×10**^**−6**^	**7.0 ± 0.5 ×10**^**−7**^	**4.4 ± 3 ×10**^**−7**^	**9.0 ± 2 ×10**^**−7**^
	*C*. *freundii* [pBR322]	*2*.*9 ± 0*.*4 ×10*^*−7*^	*2*.*3 ± 0*.*7 ×10*^*−7*^	*2*.*5 ± 1*.*4 ×10*^*−8*^	*1*.*6 ± 0*.*6 ×10*^*−6*^	*1*.*8 ± 0*.*6 ×10*^*−6*^
		**< 1.8 ×10**^**−9**^	**4.2 ± 4.2 ×10**^**−9**^	**5.0 ± 2.7 ×10**^**−8**^	**1.2 ± 0.6 ×10**^**−6**^	**1.3 ± 1 ×10**^**−7**^
	*S*. *enteritidis* [pBR322]	*6*.*4 ± 0*.*7 ×10*^*−9*^	*9*.*0 ± 1*.*3 ×10*^*−8*^	*1*.*7 ± 1*.*2 ×10*^*−8*^	*1*.*3 ± 0*.*5 ×10*^*−6*^	*6*.*0 ± 1 ×10*^*−6*^
		**< 1.6 ×10**^**−9**^	**2.7 ± 1.4 ×10**^**−8**^	**8.6 ± 5.8 ×10**^**−8**^	**5.0 ± 2.5 ×10**^**−8**^	**4.9 ± 0.7 ×10**^**−7**^

The frequency of F-plasmid conjugal transfer is given in boldface. Mobilization frequency/conjugal transfer frequency is the number of transconjugants per recipient. Shown are mean values from 3 independent experiments, and standard deviations are indicated.

### EcoVIII RM system can induce plasmid DNA release from *E*. *coli* cells

**Fig 3:**
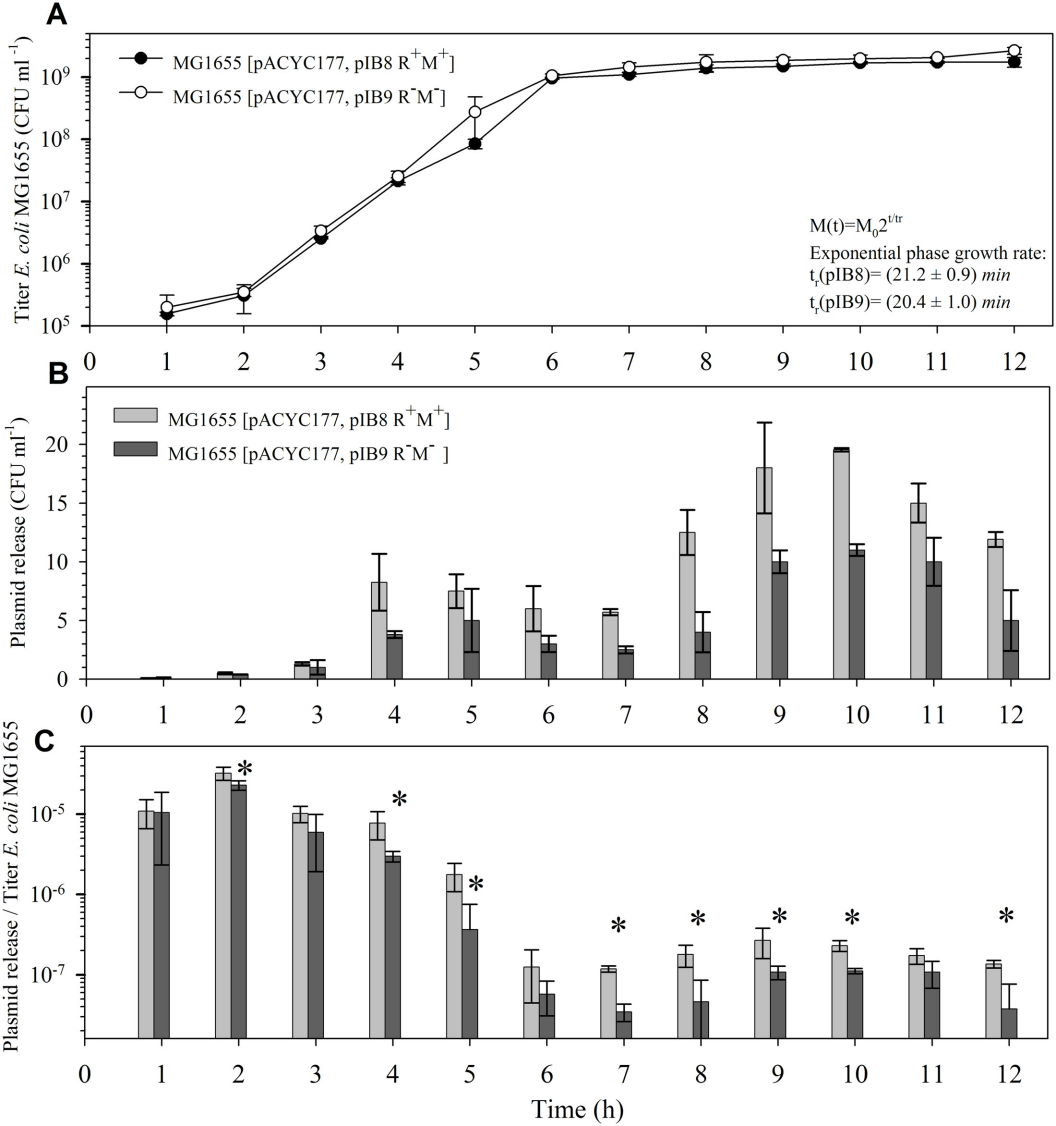
Effect of the EcoVIII RM system on plasmid release. Growth curves of *E*. *coli* MG1655 [pIB8, pACYC177] and MG1655 [pIB9, pACYC177] (**A**). Plasmid release indicated as number of pACYC177-positive transformants (**B**). The efficiency of plasmid release was determined as a ratio of transformants against the titer of the host (**C**). Each column represents the mean value (± standard deviation) from three repeats. Statistically significant differences in plasmid release as analyzed by Student’s *t* test, indicated by asterisk, were observed between bacteria carrying pIB8 (EcoVIII R^+^M^+^) and those that carried pIB9 (EcoVIII R^−^M^−^) (*P*<0.05; Student *t* test).

Many bacteria release their genomic DNA to the extracellular matrix [48, 49]. This makes an enormous pool of genetic determinants which, when acquired, can increase the microbial genetic diversity. In this work, we investigated an effect of the EcoVIII RM system on plasmid DNA release from bacterial cells. It was shown already that stable maintenance of genetic elements that carry the RM systems is dependent on molecular mechanisms based on postsegregational cell killing [29, 30]. In our experiment, we used *E*. *coli* MG1655 carrying plasmid pIB8 (EcoVIII R^+^M^+^) and pACYC177. In a control experiment the same strain was carrying either the pIB9 (EcoVIII R^−^M^−^) and pACYC177 or pACYC177 plasmid alone. Plasmid release was monitored by an assay based on *E*. *coli* transformation. The results obtained are shown in Fig 3. First, there was no difference in the growth rate between bacteria with pIB8 (EcoVIII R^+^M^+^) and those with pIB9 (EcoVIII R^−^M^−^) (Fig 3A). Samples of bacterial cultures were taken and analyzed for the presence of released plasmid DNA (pACYC177). Prior to analysis, samples collected at 1 h intervals were centrifuged and filtered through nitrocellulose filter to remove remaining bacterial cells. The absence of viable bacteria was verified by plating samples on LB agar plates. This procedure proved to be effective and no bacteria were isolated from the filtered samples. Then, plasmid DNA was precipitated and used for transformation of *E*. *coli* HB101 competent cells prepared using the CaCl_2_ standard method [34]. We have found that plasmid DNA release, indicated by the number of pACYC177-positive transformants (Ap^R^, Km^R^) was observed through all phases of bacterial growth (Fig 3B). Statistical analysis revealed a positive correlation between presence of a plasmid with EcoVIII RM system (pIB8) and release of plasmid DNA from *E*. *coli* cells at expotential and stationary-phase of growth (Fig 3C, *P*<0.05; Student *t* test).

### Bacteria can acquire pEC156-derivatives by means of natural transformation

**Fig 4:**
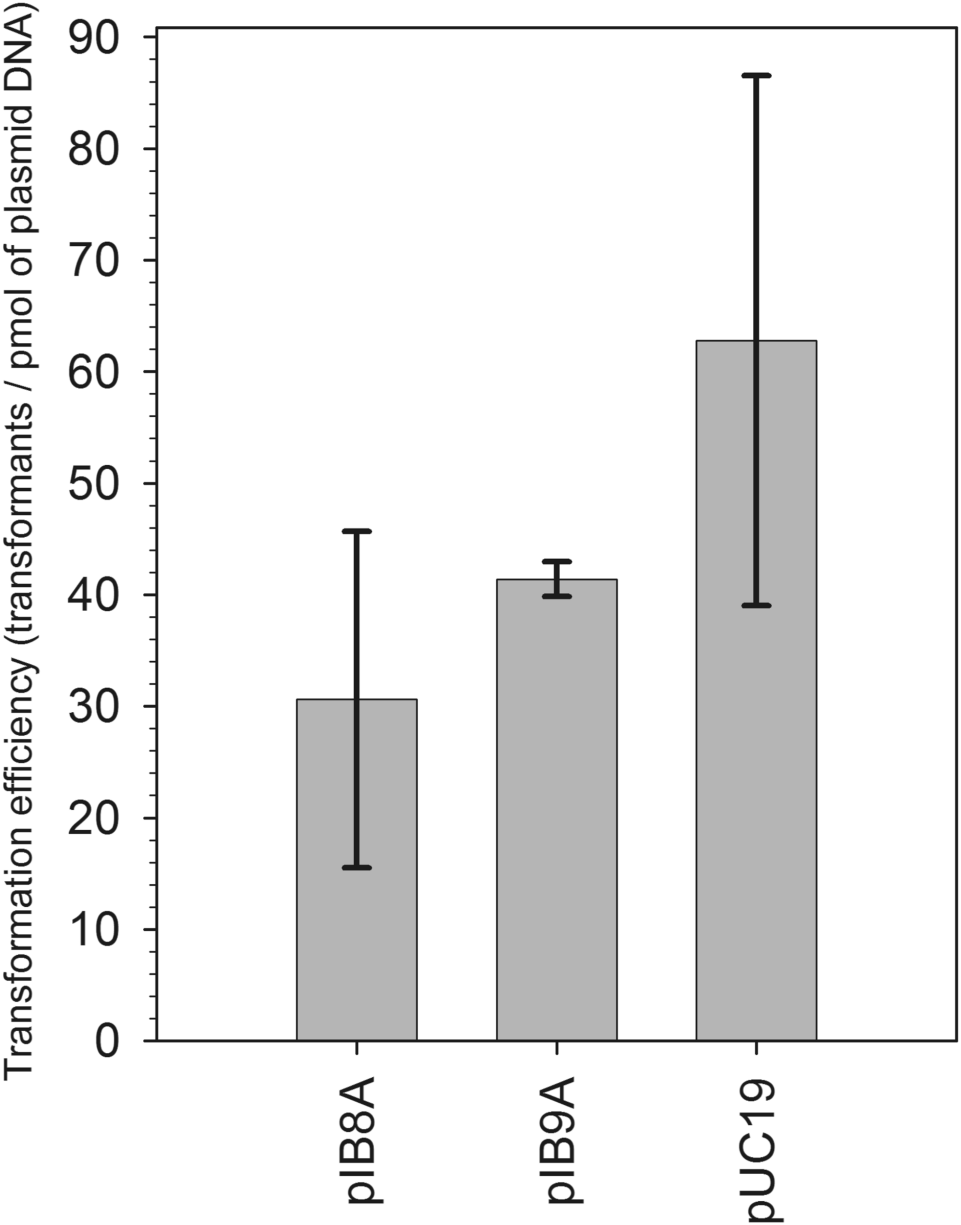
Efficiency of natural transformation of *E*. *coli* HB101 with pEC156-derivatives pIB8A (EcoVIII R^+^M^+^) and pIB9A (EcoVIII R^−^M^−^). Plasmid pUC19 was used as a control. Each column represents the mean value (± standard deviation) from three repeats. Statistical significance analyzed by Student’s *t* test revealed lack of correlation between presence of EcoVIII RM system and efficiency of natural transformation (*P* = 0.12; Student *t* test).

It was demonstrated by others that free DNA released to the environment can be acquired by some bacteria due to their capacity for natural transformation [50, 51]. According to the analysis reported recently RM systems are over-represented in naturally competent microorganisms [14]. This can explain the presence of a large number of diverse RM systems in bacteria belonging to genus *Helicobacter* or *Neisseria* that show a natural ability to uptake DNA from the environment [52, 53]. To introduce the pEC156-derivatives pIB8/pIB9 into *E*. *coli* cells we used a method developed by Sun et al. [42]. In this method there is no need to treat bacteria with any chemical agent (e.g. CaCl_2_) or temperature shifts. Simply, plasmid DNA is mixed with sample of an *E*. *coli* culture cultivated without agitation in LB medium at 37°C until it reached stationary-phase. For this experiment, we modified the pIB8 and pIB9 plasmids by replacing the gene conferring resistance to chloramphenicol with a cassette carrying β-lactamase gene (Ap^R^) as described under Materials and methods. This was necessary, as protein synthesis is a prerequisite to natural transformation [42]. As a host, *E*. *coli* HB101 was used, that exhibits the highest natural transformability among laboratory strains [42]. The results are shown in Fig 4. We found that the efficiency of *E*. *coli* natural transformation with pIB8A (EcoVIII R^+^M^+^) was similar to pIB9A (EcoVIII R^−^M^−^). For 1 pmol of plasmid DNA used in the experiment, we obtained 30±15 transformants for pIB8A. In the case of control experiments with the pIB9A and pUC19 plasmids, we obtained 41±2 and 63±24 transformants/pmol, respectively. Statistical significance analysis with the use of Student’s *t* test revealed lack of correlation between presence of EcoVIII RM system on the plasmid and efficiency of natural transformation (*P* = 0.12; Student *t* test).

## Discussion

The objective of the report presented here was to investigate the routes of transmission of pEC156 derivatives (R^+^M^+^/R^−^M^−^), a naturally occurring plasmid of *E*. *coli* E1585-68 that carries genes of the EcoVIII RM system. Specifically, we were focused on natural transformation and mobilization by conjugative plasmids. In this respect, an important issue is the host range which in the case of pEC156 is determined by plasmid and host-encoded functions. We have shown previously that pEC156 possesses a ColE1-type replicon [16]. Replication of plasmids of this kind relies on host-encoded enzymes: DNA polymerase I, RNA polymerase, RNaseH, DNA gyrase, topoisomerase I, as well as proteins involved in the primosome formation: PriA, PriB, PriC, DnaB, DnaC, DnaG and DnaT [54]. These specific protein requirements limit ColE1-type plasmids’s host range mostly to the members of *Enterobacteriaceae* family [55]. However, there are reports that bacteria not related to this family, such as *Legionella pneumophila* and *Shewanella baltica*, can also sustain replication and maintenance of ColE1-like plasmids [56, 57]. In our previous work we have shown that derivatives of pEC156 can be propagated in enterobacteria such as: *Serratia marcescens*, *Enterobacter cloacae*, *Klebsiella oxytoca*, *Salmonellas enteritidis* and *Proteus vulgaris* [28]. In these bacteria stable maintenance of pEC156 is ensured by the cis-acting *cer* site involved in resolution of plasmid multimers, as well as by plasmid-encoded gene for EcoVIII endonuclease [28].

The pEC156-derivatives used in our study can be mobilized by *E*. *coli* conjugative plasmids F (IncFI) and R64 (IncI1) due to the presence of two origins of transfer that are the targets for specific relaxases that are the key proteins in conjugation. The host range of these self-transmissible plasmids is rather narrow as their replication and maintenance capacity is dependent on host enzymes, as well as *cis* and *trans* acting plasmid determinants [58, 59]. These elements can form a functional network only in some bacteria, restricting the transfer of aforementioned conjugal plasmids, as well as mobilization of non-self transmissible ColE1-type plasmids, to members of the *Enterobacteriaceae* family [59–62]. In our work, we performed single-species and heterospecific matings between selected members of the *Enterobacteriaceae* family that show close phylogenetic relationship. In some hosts, we noted a striking difference in frequency of pEC156-derivative transfer with respect to functionality of the EcoVIII RM system. The pIB8 (EcoVIII R^+^M^+^) was mobilized less efficiently when compared to pIB9 deprived of the RM system. This indicates that presence of RM system may limit the spread of the native plasmid among enterobacteria. In some cases, we observed mobilization of pEC156-derivative but not self transfer of the F-plasmid. This was most evident in the case of *E*. *coli-S*. *enteritidis* matings (Table 2). The reason for this might be rooted in the toxicity of the RM system itself, as well as in conditions under which our experiment was performed. It was shown that optimal frequency of F-plasmid conjugal transfer in *Salmonella* and *E*. *coli* requires filter matings on plates with minimal medium (M9 agar plates, [62]). In our standard protocol, matings were performed in rich liquid medium (Luria broth). Conditions of this kind result in 400-fold reduction in efficiency of F-plasmid transfer [63]. On the other hand, extensive analysis of plasmid profiles of *Salmonella* strains revealed in the case of *S*. *enteritidis* isolates abundance of ColE1-like plasmids with mobilization capacity and apparent absence of conjugative plasmids [64].

We also investigated the possibility of spontaneous acquisition of pEC156-derivatives by bacteria. There are several independent studies that report on the ability of *E*. *coli* cells to uptake plasmid DNA [42, 65–68]. In our work, we used the protocol that neither involved treatment of *E*. *coli* cells with CaCl_2_ nor heat shock which could induce plasmid DNA uptake [42]. After testing several laboratory strains (MG1655wt, DH5α and HB101) we concluded that most satisfactory results were obtained with the HB101 strain. As this strain shows F^-^ phenotype, it is unlikely that plasmid DNA uptake in this procedure is linked to the so called classical DNA take-up machinery that involves type IV pili and which is characteristic for such model organisms like *Haemophilus influenzae*, *Neisseria gonorrhoeae* or *Vibrio cholerae* [69–71].

We also found, that bacteria possessing the EcoVIII RM system can release plasmid content to the environment. This can be explained by self-restriction of genomic DNA which is either the effect of incidental lack of balance for enzymes constituting the RM system [72] or postsegregational killing of cells that lost the plasmid that carry the RM system [73]. In our laboratory we have found that restriction activity of EcoVIII RM system can be affected by Mg^2+^ ions that are among the most abundant ions in a bacterial cell. We have shown that Mg^2+^, at a physiological concentration (1–2 mM), is a strong inhibitor of EcoVIII methyltransferase (IC_50_ 2 mM Mg^2+^) but not of EcoVIII endonuclease [74]. This means that inside bacterial cells, the activity of M.EcoVIII may be inhibited while activity of EcoVIII restriction endonuclease is not affected. This may shift the balance in functioning of the RM system towards restriction. Degradation of genomic DNA may lead to death of the cell and release of genomic DNA content. Similar effects were observed in case of bacteria carrying EcoRI RM system [72, 73], as EcoRI DNA methyltransferase is also inhibited by Mg^2+^ [75]. It was shown that after loss of the plasmid that carried EcoRI RM system, substantial portion of cells underwent morphological changes. First, long filaments were observed which is characteristic for the SOS response, followed by anucleation and death of the cells [73].

**Fig 5:**
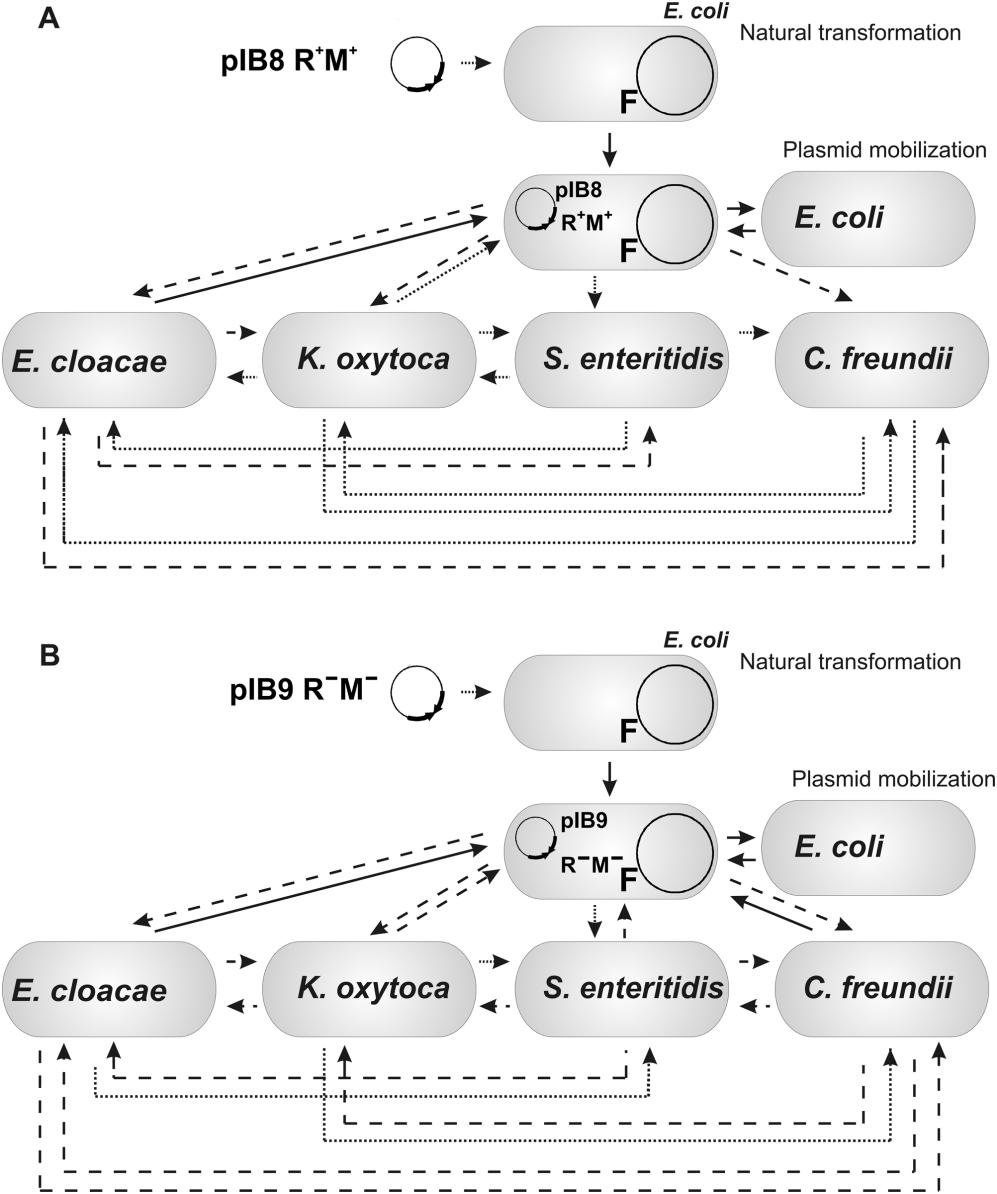
Mobility of pEC156-derivatives pIB8 EcoVIII R^+^M^+^) (A) and pIB9 (EcoVIII R^−^M^−^) (B) among enterobacteria. Arrows indicate direction of plasmid dissemination and rate of mobilization frequency based on experimental data presented in Tables 2 and 3 [solid line, high frequency (10^−3^–10^−5^); dashed line, medium frequency (10^−6^–10^−7^); and dotted line, low frequency (10^−8^–10^−9^)].

The obtained results allowed us to propose possible routes of transmission of pEC156-derivatives as outlined in Fig 5. We have demonstrated that pEC156-derivatives can be effectively transferred among enterobacteria by means of plasmid mobilization and natural transformation. Out of these, we found that mobilization by conjugal plasmid is a more efficient way to spread pEC156-derivatives among enterobacteria. Such dissemination of the plasmid-borne RM systems may promote formation of a barrier in bacteria that modulates exchange of genetic determinants and in consequence results in differentiation of bacterial lineage into distinct microbial subpopulations [76–78] that are better adapted to the particular ecological niche [79, 80]. Comparative analyses of sequenced genomes supports the idea that ecological factors strongly affect gene transfer and as such they contribute to shaping the structure of bacterial and archeal populations [81–83].

## Supporting Information

S1 Table The scheme of pEC156-derivatives mobilization experiment. (DOC)
