# Supplementary material for: Plasmid pEC156, a Naturally Occurring Escherichia coli Genetic Element That Carries Genes of the EcoVIII Restriction-Modification System, Is Mobilizable among Enterobacteria
Source: PLoS One. 2016 Feb 5;11(2):e0148355. doi: 10.1371/journal.pone.0148355 (PMC4743918; doi:10.1371/journal.pone.0148355)
Supplement: S1 Table — (DOC) [file pone.0148355.s001.doc]

**S1 Table.** The scheme of pEC156-derivatives mobilization experiment.

| Donor |  | Recipient |  | Transconjugantsrelevant features | Phenotype |
| --- | --- | --- | --- | --- | --- |

| *E. coli* DH5α Rif [F’*,* pIB8/pIB9] | X | *E. coli* HB101 [pBR322]  *E. coli* MG1655∆*lac* [pBR322]  *E.* *cloacae* [pBR322]  *K.* *oxytoca* [pBR322]  *C. freundii* [pBR322]  *S. enteritidis* [pBR322] | → | Transconjugant [pIB8/pIB9, pBR322]  Transconjugant [F’, pBR322] | ATC  ATK |
| --- | --- | --- | --- | --- | --- |
|  |  |  |  |  |  |
| *E.* *cloacae* [F’*,* pIB8/pIB9] | X | *E. coli* HB101 [pBR322]  *E. coli* MG1655∆*lac* [pBR322]  *E.* *cloacae* [pBR322]  *K.* *oxytoca* [pBR322]  *C. freundii* [pBR322]  *S. enteritidis* [pBR322] | → | Transconjugant [pIB8/pIB9, pBR322]  Transconjugant [F’, pBR322] | ATC  ATK |
|  |  |  |  |  |  |
| *K.* *oxytoca* [F’*,* pIB8/pIB9] | X | *E. coli* HB101 [pBR322]  *E. coli* MG1655∆*lac* [pBR322]  *E.* *cloacae* [pBR322]  *K.* *oxytoca* [pBR322]  *C. freundii* [pBR322]  *S. enteritidis* [pBR322] | → | Transconjugant [pIB8/pIB9, pBR322]  Transconjugant [F’, pBR322] | ATC  ATK |
|  |  |  |  |  |  |
| *C. freundii* [F’*,* pIB8/pIB9] | X | *E. coli* HB101 [pBR322]  *E. coli* MG1655∆*lac* [pBR322]  *E.* *cloacae* [pBR322]  *K.* *oxytoca* [pBR322]  *C. freundii* [pBR322]  *S. enteritidis* [pBR322] | → | Transconjugant [pIB8/pIB9, pBR322]  Transconjugant [F’, pBR322] | ATC  ATK |
|  |  |  |  |  |  |
| *S.* *enteritidis* [F’*,* pIB8/pIB9] | X | *E. coli* HB101 [pBR322]  *E. coli* MG1655∆*lac* [pBR322]  *E.* *cloacae* [pBR322]  *K.* *oxytoca* [pBR322]  *C. freundii* [pBR322]  *S. enteritidis* [pBR322] | → | Transconjugant [pIB8/pIB9, pBR322]  Transconjugant [F’, pBR322] | ATC  ATK |

C – chloramphenicol, A – ampicillin, T – tetracycline, K –kanamycin; *E. cloacae* refers to *Enterobacter* *cloacae*
